# Vertically-aligned graphene flakes on nanoporous templates: morphology, thickness, and defect level control by pre-treatment

**DOI:** 10.1088/1468-6996/15/5/055009

**Published:** 2014-10-31

**Authors:** Jinghua Fang, Igor Levchenko, Shailesh Kumar, Donghan Seo, Kostya (Ken) Ostrikov

**Affiliations:** 1 CSIRO Manufacturing, PO Box 218, Lindfield, NSW 2070, Australia; 2 School of Physics, University of Melbourne, Parkville, VIC 3010, Australia; 3 Plasma Nanoscience, School of Physics, The University of Sydney, Sydney, NSW 2006, Australia; 4 Institute for Future Environments and School of Chemistry, Physics, and Mechanical Engineering, Queensland University of Technology, Brisbane, QLD 4000, Australia

**Keywords:** graphene flakes, nanotemplates, plasma treatment

## Abstract

Various morphologies of the vertically-aligned graphene flakes were fabricated on the nanoporous templates treated with metal ions in solutions, as well as coated with a thin gold layer and activated in the low-temperature Ar plasma. The thickness and level of structural defects in the graphene flakes could be effectively controlled by a proper selection of the pre-treatment method. We have also demonstrated that various combinations of the flake thickness and defect levels can be obtained, and the morphology and density of the graphene pattern can be effectively controlled. The result obtained could be of interest for various applications requiring fabrication of large graphene networks with controllable properties.

## 1. Introduction

One-and two-dimensional nanostructures possess many unique properties such as very high mechanical strength, chemical stability, and very large surface areas [1, 2]. Carbon nanowalls and vertically-aligned graphene flakes are among the most promising two-dimensional materials potentially useful for many important applications [3, 4]. They are important for many applications such as optoelectronics, nanoelectronics, sensing devices, energy and signal conversion and many other applications [5, 6].

One of the techniques commonly used for the production of vertically-aligned carbon nanowalls is a simple and cheap chemical vapour deposition (CVD) method [7], which still lacks effective control of the process. The nanostructures grown by the CVD techniques are often of low quality, and their morphology cannot be changed because of the limitations intrinsic to the CVD method. On the other hand, plasma-based [8, 9] and laser-based [10] methods are more controllable due to the many process parameters (such as plasma density, ion and electron energy, voltage applied etc) affecting the developing structure [11, 12]. The structure and morphology of the networks of carbon nanotubes and nanowalls grown by plasma-based methods demonstrate better controllability and diversity [13, 14]. When growing the vertical networks on the nanoporous substrates, which are very promising for various special applications such as micro-fluidics and sensors requiring gas penetration through the substrates, the problem of structure control becomes even harder and new methods of the network morphology control are required.

Here we report on a novel inductively-coupled plasma (ICP) based approach to grow the networks of vertically-aligned few-walled graphene flakes (vertically-aligned carbon nanowalls) on nanoporous alumina templates. To effectively control the morphology, thickness and level of the structural defects of graphene flake networks, various methods of pre-treatment were used including noble-gas plasma and solution-based treatments with various ions (Co^+^, Pd^+^, Au^+^). Also, a continuous gold film was applied onto one of the substrates to control the network morphology. The results of our experiments demonstrate a strong dependence of the carbon nanowalls network morphology on the pre-treatment technique.

## 2. Experimental details

**Figure 1.**
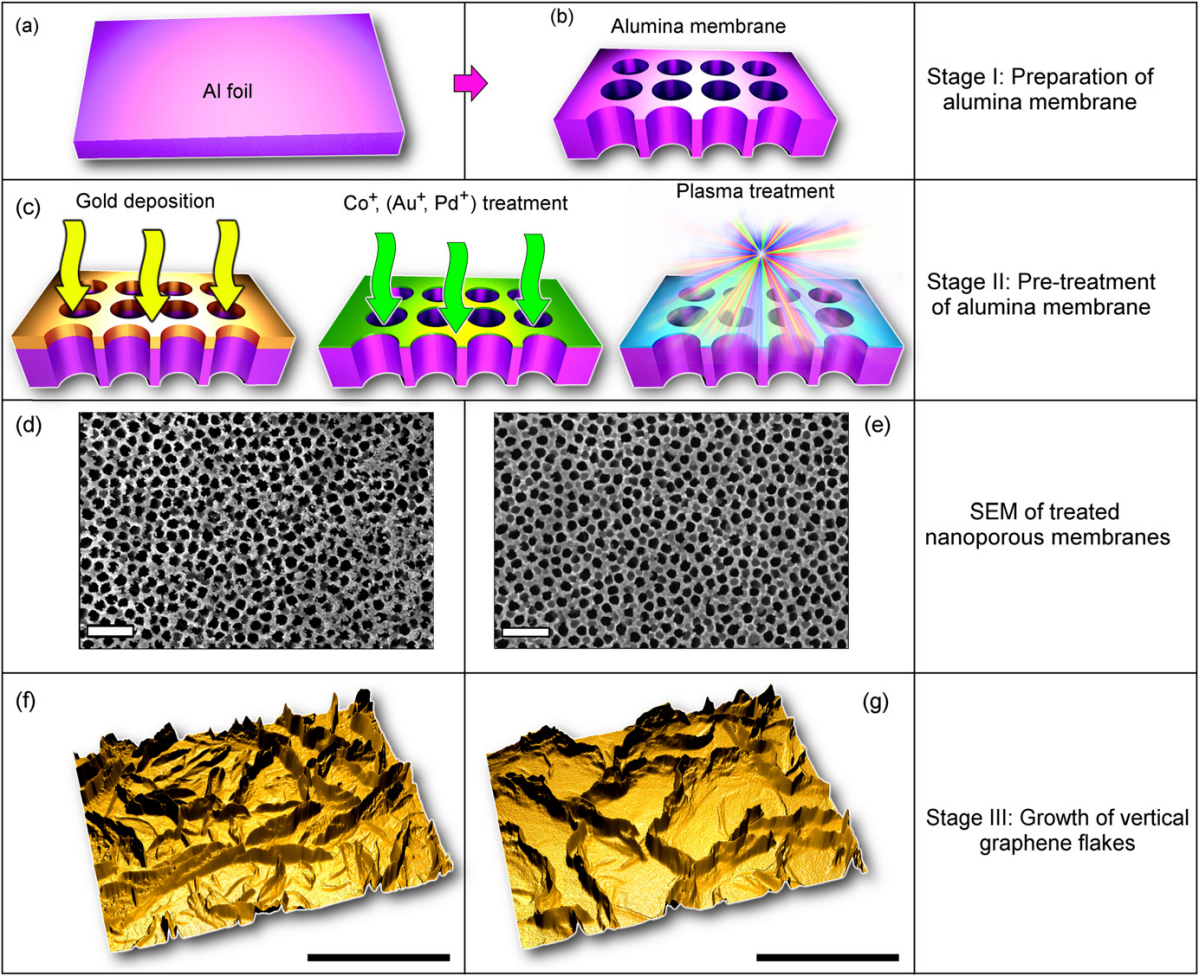
Schematic of the process. This Al foil (a) was first anodized to produce the nanoporous template (b). At Stage II (c), the samples (nanoporous templates) were subjected to various pre-treatment procedures (gold deposition, ion treatment in solutions, and ICP plasma treatment in Ar. Pd ion-treated (d) and Ar plasma-treated (c) samples exhibit different surface morphologies. At Stage III, the vertically-aligned carbon nanowall networks of various morphologies were grown in ICP plasmas ignited in a methane–hydrogen mixture. Three-dimensional models of the carbon nanowall networks reconstructed using the half-tone SEM images (f), (g). Scale bars are 1000 nm.

The process starts from fabrication of alumina (Al_2_O_3_) templates using a simple and convenient anodization technique [15]. In this work we have chosen a quite standard process based on the most popular oxalic acid (0.4 M) as electrolyte [16] which guarantees fabrication of the template with the target features, i.e., with pore sizes of about 100–200 nm (i.e., significantly smaller than the size of graphene flakes reaching several *μ*m). The anodization of the Al foil (thickness of about 300 *μ*m) was conducted under direct current conditions, with the dc voltages of 40–60 V and electrolyte temperature of about 0 °C to achieve highly-ordered arrays [16]. The anodization voltage and acid concentration were chosen by taking into account that both the channel diameter and the step between the channels increase with the anodization voltage [17], whereas an increase in the acid concentration results in the decrease of the inter-channel distances [18]. Each sample was a disk with a diameter of about 15 mm. The schematic of this process is shown in figure 1, more details of this process and properties of the nanoporous templates can be found elsewhere [19]. The ready nanoporous alumina templates are perforated with straight channels. These templates (pore size of 100–200 nm) were then subjected to various types of pre-treatment (figure 1(c)). Firstly, one sample (nanoporous template) was not treated at all before the nanowalls growth (reference sample). Second, one sample (nanoporous template) was treated in ICP plasmas under the following conditions: Ar flow—10 sccm, chamber pressure—2.0 Pa, radio-frequency (RF) power used to ignite plasma750 W, and treatment time—2 min Next, the three samples were immersed for 24 h into the 0.01 M KAu(CN)_2_, 0.1 M CoSO_4_, and 0.1 M PdCl_2_ solutions, respectively. Scanning electron microscopy (SEM) images of the samples pre-treated using Pd ions and Ar plasma are shown in figures 1(d) and (e), respectively. The treated samples were dried with a jet of dry nitrogen gas. Finally, one sample was put into the AJA sputter coating system and coated with a 30 nm Au thin film at a deposition rate of 0.2 nm s^−1^. The gold-coated sample exhibited a continuous gold layer before the final plasma-based growth, and this layer fragmented into islands of 10–15 nm in size during the graphene growth in the CH_4_/Ar/H_2_ plasma described below. The samples treated in KAu(CN)_2_, CoSO_4_, and PdCl_2_ solutions did not show any observable changes.

The sample treated by the Ag ICP plasma featured slightly sharpened edges of the channels. It was demonstrated for the case of silicon substrates that such sharp edges can contribute to the nucleation and growth of carbon nanotubes and graphene flakes [20]. Moreover, such nano-hillocks can catalyse the nanowires [19]. Besides, formation of Al reduced by the preferential hydrogen removal due to the alumina template bombardment by heavy Ag ions is also possible.

After that, all six samples were installed in the ICP chamber, and the network of vertically-aligned graphene flakes was grown under the following conditions (the same for all samples treated): CH_4_/Ar/H_2_ ratio—2 : 1 : 1, chamber pressure—3.0 Pa, RF power—850 W (power density in the discharge chamber of about 0.5 W cm^−3^), RF frequency 13.56 MHz, treatment time—6 min. Probe measurements show the following plasma parameters in the discharge: plasma density of about 10^12^ cm^−3^, electron energy of 2–3 eV [14]. The plasma parameters were not varied in this work, since the effect of the pre-treatment was the main focus of the study.

After deposition, samples were characterized using SEM, transmission electron microscopy (TEM) and Raman technique. To better characterize the morphology and structure of the graphene flake arrays, we have reconstructed the three-dimensional models using the half-tone SEM images (see figures 1(f) and (g)). The area density of the flake network morphology was examined by calculating the projected flake areas.

## 3. Results and discussion

**Figure 2.**
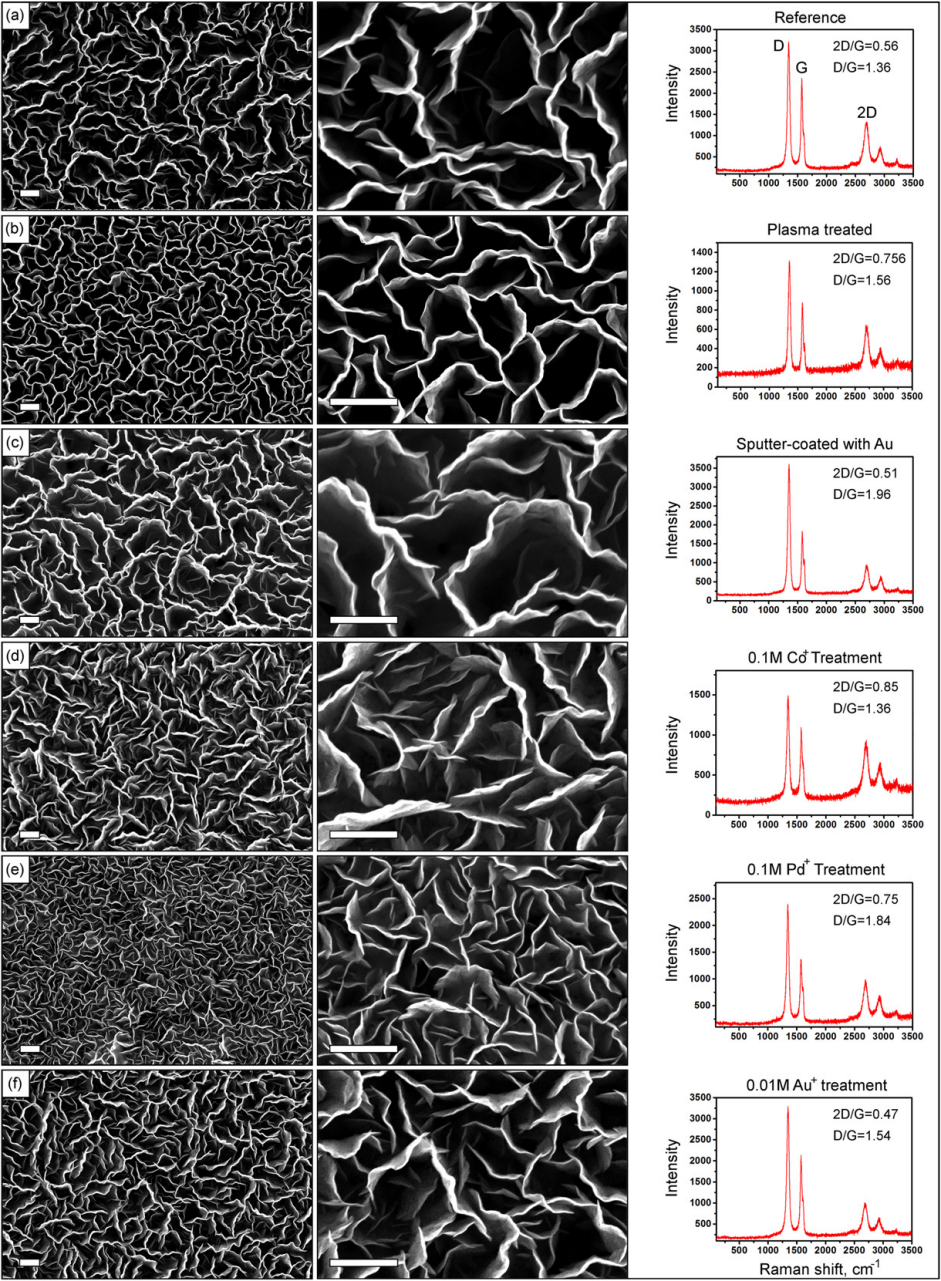
Low- and high-resolution SEM images and Raman spectra of the vertical graphene nanowalls grown on nanoporous templates after different pre-treatments. Scale bars are 500 nm.

Samples were observed with a field-emission scanning electron microscope (FE-SEM, type Zeiss Auriga) operated at electron beam energy of 1–5 keV with an InLens secondary electron detector. Figure 2 illustrates the results of SEM and Raman characterization of all six samples grown on the nanoporous templates after various treatment processes. From these images one can clearly see that the morphologies of the nanowalls network grown on various samples are significantly different. Indeed, the nanowalls density is apparently higher for the plasma-treated and Pd^+^-treated samples.

Micro-Raman analysis was performed using a Renishaw inVia spectrometer with laser excitations of 514 and 633 nm at a spot size of ∼1 *μ*m^2^. To enhance the accuracy of measurements, Raman spectra from multiple spots of the samples were collected and averaged. Raman spectra for all the samples are also shown on figure 2. Normally, the sharp peak appearing around 1580 cm^−1^ in the Raman spectrum of graphene is the G band, i.e., the in-plane vibrational mode that involves sp^2^ hybridized carbon atoms which comprise the graphene sheet. Other major bands (D at approximately 1328 cm^−1^ and 2D at approximately 2687 cm^−1^) were also found in the Raman spectra. Importantly, ratio of 2D and D bands indicates the number of layers of graphene. The ratio is larger for thinner flakes containing fewer graphene layers and smaller for thick flakes [21]. In our experiment, the thinnest graphene was obtained on the sample treated with 0.1 M CoSO_4_ (Co^+^ ions) for 24 h at a room temperature, the next are the plasma-treated sample and the sample treatedby the 0.1 M PdCl_2_ for 24 h at a room temperature. However, the thicker sample was grown on the substrate treated by 0.01 M KAu(CN)_2_, i.e., Au^+^ ion treatmentpromotes formation of many graphene layers. The reference sample, as well as samples coated with a 30 nm Au film, has demonstrated a largegraphene layer number.

The summary of the Raman bands for all the samples studied is provided in table 1.

**Table 1.** G, D, and 2D band positions for different treatment processes.

Sample	D/G band ratio	2D/G band ratio
Reference	1.36	0.56
Plasma treated	1.56	0.756
Au^+^ treated	1.54	0.47
Co^+^ treated	1.36	0.85
Coating with Au (30 nm)	1.96	0.51
Pd^+^ treated	1.84	0.75

Another very important factor to quantify the quality of graphene is the peak intensity ratio of D and G bands. In this case, the larger ratio indicates the presence of defects in the fabricated graphene. In our experiments the sample treated by CoSO_4_ solution has demonstrated the much lower levels of defects than other samples. Use of 0.01 M KAu(CN)_2_ solution to treat the template results in more defect. Coating the sample with Au film resulted in more defects in the graphene structure.

The summary of the sample treatments and characteristics is provided in table 2.

**Table 2.** Summary of the sample treatments, thickness and defect levels.

Sample	Treatment	Thickness (from 2D/G ratio)	Defect levels (from D/G ratio)
Reference	No treatment	Thick	Lowest
Plasma treated	Ar plasma 750 W, 2 min	Thin	Low
Au^+^ treated	0.01 M KAu(CN)_2_	Thickest	Low
Co^+^ treated	0.1 M CoSO_4_	Thinnest	Lowest
Coating with Au	Sputtering, 30 nm	Thick	Highest
Pd^+^ treated	0.1 M PdCl_2_	Thin	High

**Figure 3.**
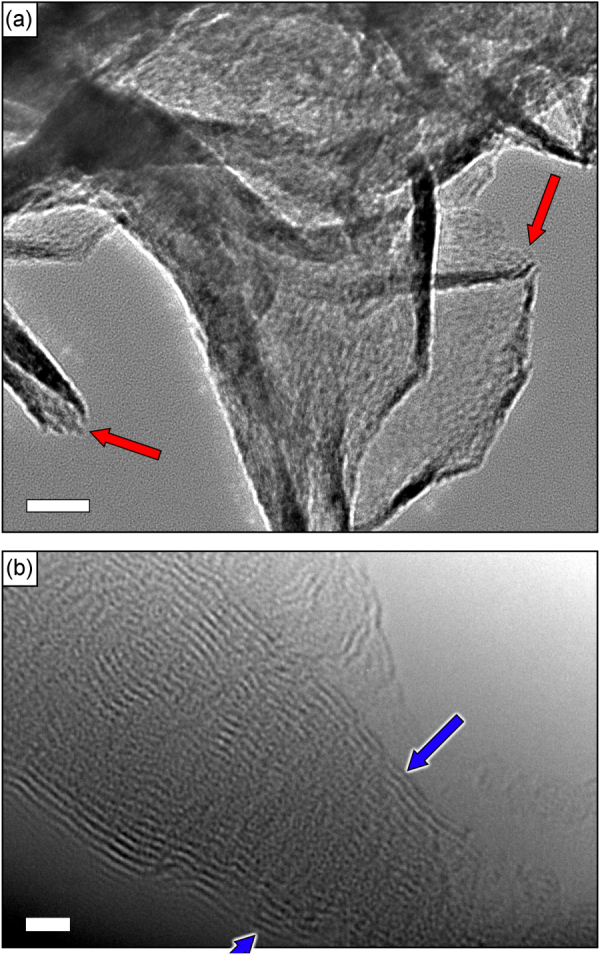
Low- and high-resolution TEM images of the graphene flake edges. Red arrows show the open graphitic edges (a). Each flake consists of 10−20 layers (b). Scale bars are 20 (a) and 2 (b) nm.

**Figure 4.**
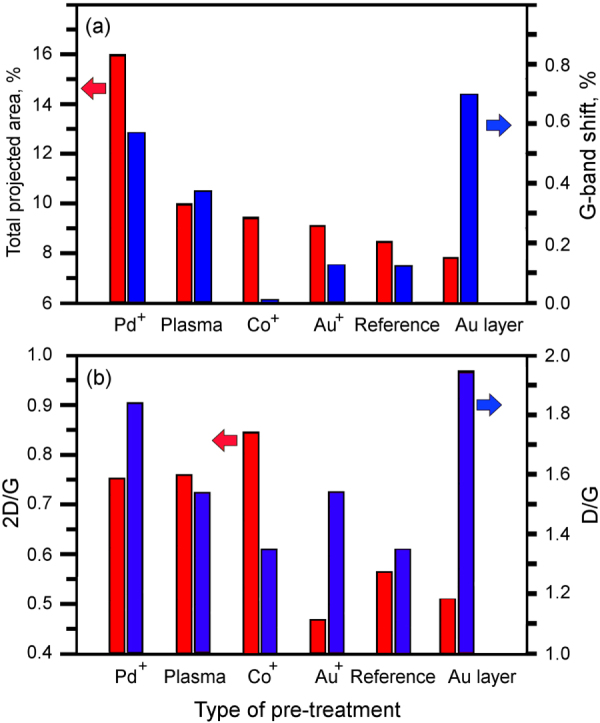
Dependence of total graphene flake projected area, G-band shift, and 2D/G and D/G shifts of the graphene flakes on pre-treatment. The total projected area is highest for the Pd^+^-treated samples which demonstrate the highest network density on SEM images (figure 2).

To additionally characterize the graphene structure, the grown flakes were observed with a JEOL 2100 TEM. An electron beam energy of 200 keV was used for the analysis. TEM analysis has shown that the flakes consist of several (up to 10–20) single graphene layers, see figure 3. Open graphitic edges (shown with arrows in figure 3(b)) were also found on TEM images. Bar distributions of the sample parameters such as total nanowall projected area, G-band shift, and 2D/G and D/G shifts are shown in figure 4.

**Figure 5.**
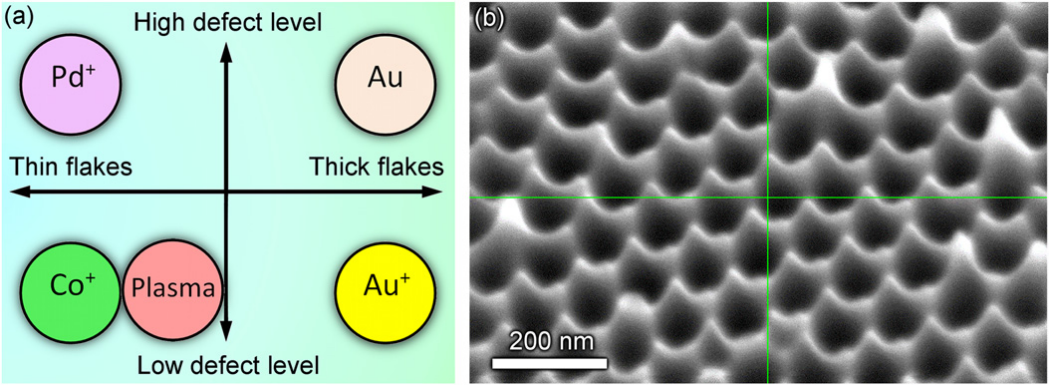
Pre-treatment ensures all basic combinations of the thickness/defect level of the vertical graphenes grown on the nanoporous alumina templates (a). Very sharp edges of the nanoporous alumina membrane (SEM tilted view) (b).

Analysis of the distributions of graphene flake thickness and defect levels in the six fabricated samples reveals a very important observation, namely fabrication of graphene flake pattern with virtually any combination of the thickness/defect level characteristics is possible using the techniques of pre-treatment proposed here. Indeed, from table 2 one can see that the *thickest* sample (Au^+^ treated) demonstrates the *low* defect level, whereas the *thinnest* sample (Co^+^ treated) demonstrates *the lowest* level of the defects. On the other hand, Pd^+^ treated sample is *thin* and simultaneously, it has *high* level of the crystalline structure defects; and vice verse, the sample coated with 30 nm gold layer is *thick* and demonstrates the *highest* level of the structural defects (see figure 5(a)).

It should be noted that both thickness and defect level are among the most important graphene flake characteristics, since they directly determine the mechanical strength and magnetic properties of the graphene. Raman measurements show that the defect level in our samples does not exceed 10^13^ cm^−2^, i.e. ≈1% [22]. This level is rather low, the mechanical properties of the graphenes do not change significantly with the defect (vacancies) level reaching 5% [23]. Thus, the level of defects in our samples is low enough to not significantly compromise the mechanical properties.

On the other hand, the defect level is very important to control the magnetization in graphene. Indeed, the magnetism in the graphene flakes depend mainly on the defect level (due to the formation of ferromagnetic ordering in the sublattices) and edge states [24]. Apparently, the treatment we used here ensures efficient control of the defects in the graphene, and simultaneously, the control of the new morphology (see figure 2) and hence, control over the length and states of the flake edges. The measured magnetic moment was found to be proportional to the density of vacancies, and the significant magnetic moment appears when the defect level reaches 10^12^–10^13^ cm^−2^ [25, 26]. This means that the defect level of our samples is low enough (even though the plasma-induced ion bombardment is often considered as a ‘harsh growth condition’) to not compromise the mechanical properties, yet high enough to ensure a significant magnetic moment, i.e., the obtained magnetic moment may be interpreted as somewhat optimal.

The edges of the nanoporous template are also an important growth factor which is responsible for the graphene nucleation and, on the other hand, determine the vertical graphene flake pattern morphology via attachment of various ions (Co^+^, Au^+^, Pd^+^) during the pre-treatment. We have conducted several experiments attempting to grow vertically-aligned graphene flakes (note that in this work we focused on the *vertical nanowalls* which can serve as a platform for bio-sensors, energy storage devices, etc) on flat alumina substrates (and importantly, without commonly used catalysts). We have found that the treatment methods described in this work (Ar plasma pre-treatment and solution treatment) were not effective. Specifically, nucleation and growth of the vertical nanowalls on flat *alumina, silica* and *silicon* surfaces were observed only on (1) significantly structured (texturized) surfaces like nanograss array [27] or (2) on catalysts. Note that in this work we were specifically interested in the formation of vertical nanowall arrays on the nanoporous substrates, potentially suitable for applications such as micro-fluidics and sensors requiring gas penetration through the substrates. Thus, we have selected nanoporous alumina as a most promising substrate which (a) ensures gas or liquid penetration, and (b) features many sharp edges.

One of the major findings described in our work is the possibility to control thickness and defect level of the carbon nanowalls by pre-treatment using different metals and methods. Let us consider some important features. Firstly, thick nanowalls with the 2D/G ratio of 0.56 were formed on the reference sample. Ar Plasma treatment resulted in significantly thinner nanowalls (2D/G = 0.75), and this could be attributed to sharpening the hole edges (see figure 5(b)). Indeed, it was already demonstrated that the array of sharp Si nanocones (similar to that shown in figure 5(b)) covered with natural silicon oxide catalysed the growth of vertical nanowalls [27]. Hence, it is quite natural to assume that similar surface profiles (with sharp edges) facilitate the nucleation and growth of nanowalls on alumina and eventually result in thinner nanowalls.

Pre-treatment using Au ions and a 30 nm thick Au coating resulted in the thickest (Au^+^) and thick (Au coating) nanowalls, which apparently could be attributed to very low carbon solubility in gold. On the other hand, the use of Co for pre-treatment resulted in the thinnest nanowalls. This is a quite expected result, since Co is one of the most popular catalysts used in the growth of carbon-based nanomaterials [28]. Nucleation of the carbon nanowalls (possible via formation of cobalt disilicide [29]) ensures formation of thin nanowalls.

The defect level of the carbon nanowalls is a function of (i) nucleation conditions, and (ii) growth conditions. Considering the details we could point out that the lowest defect layer is a feature of the Co-treated sample. As we have mentioned and referenced above, Co is an efficient catalyst for carbon-based nanostructures (nanotubes and nanowalls), so the low defect level of the nanowalls grown with Co should be considered as a quite expected result. On the other hand, the highest defect level was found in the nanowalls grown on the Au-coated (30 nm) membrane. In this case the nanowalls were nucleated on relatively thick gold nanoislands which feature low carbon solubility. Besides, the lateral field caused by the non-uniform charging of the catalyst islands could play a significant role in the nucleation of nanowalls [30]. The other methods and materials are characterized by the intermediate carbon solubility, and this could be an explanation for the intermediate properties. Besides, surface diffusion can be affected by the pre-treatment and thus contribute to the growth process [31, 32].

The substrates during the plasma-enabled growth were insulated from the chamber, and thus they were under the floating potential (several tens of volts for the used plasma parameters). Under these conditions, the ion energy is usually too low to cause any significant damage to the graphene structure.

## 4. Conclusions

In summary, we have reported on the fabrication of vertically-aligned graphene flakes of various morphologies on the nanoporous templates treated with various ions in solutions, as well as coated with a thin gold layer and activated in low-temperature Ar plasmas. We have demonstrated that various combinations of the flake thickness and defect levels can be obtained, and the morphology of the graphene pattern can be effectively controlled. The work could be of interest for various applications requiring fabrication of large graphene networks with controllable properties.
